# Preimplantation Genetic Testing for Aneuploidy Improves Cumulative Live Birth Rate and Reduces Miscarriage in Recurrent Pregnancy Loss

**DOI:** 10.1155/bmri/8875136

**Published:** 2026-03-26

**Authors:** Luping Yu, Yiqun Tang, Na Kong, Jingyu Liu, Fangfang He, Ningyuan Zhang, Jie Mei

**Affiliations:** ^1^ Reproductive Medicine Center, Nanjing Drum Tower Hospital, The Affiliated Hospital of Nanjing University Medical School, Nanjing, China, nju.edu.cn; ^2^ Drum Tower Clinic Medical College of Nanjing Medical University, Nanjing, China, njmu.edu.cn; ^3^ Department of Women′s and Children′s Health, Karolinska Institutet, Stockholm, Sweden, ki.se

**Keywords:** assisted reproduction, cumulative live birth rate, miscarriage, preimplantation genetic testing for aneuploidy, recurrent pregnancy loss, reproductive health

## Abstract

**Background:**

Recurrent pregnancy loss (RPL), defined as ≥ 2 consecutive/nonconsecutive losses, affects 1%–5% of pregnant women. This study is aimed at assessing whether preimplantation genetic testing for aneuploidy (PGT‐A) improves cumulative live birth rates and reduces miscarriage risk in couples with recurrent pregnancy loss and normal karyotypes undergoing assisted reproduction.

**Methods:**

A total of 1039 couples with recurrent pregnancy loss who underwent assisted reproductive treatment were enrolled in this retrospective cohort study between January 2013 and December 2023. Participants were stratified into two groups according to their decision to undergo PGT‐A: 200 couples who underwent PGT‐A and 839 couples who did not. In total, 670 frozen embryo transfer (FET) cycles were included in the analysis. The clinical characteristics and reproductive outcomes including the cumulative live birth rate were compared between the two groups using different statistical methods.

**Results:**

PGT‐A demonstrated significant improvements in reproductive outcomes for women with RPL. In women ≤ 35 years, PGT‐A increased the cumulative live birth rate from 51% to 70% (aRR 1.38, 95% CI 1.059–1.82; *p* = 0.025), whereas in women > 35 years, the rate improved from 21% to 35% (aRR 1.69, 95% CI 1.03–2.77; *p* = 0.037). Notably, PGT‐A also reduced miscarriage rates in younger women, with early miscarriage rates decreasing from 13% to 7% (*p* = 0.028) and late miscarriages being eliminated (0% vs. 3%, *p* = 0.017).

**Conclusions:**

Preimplantation genetic testing for aneuploidy significantly increased the cumulative live birth rate in couples with recurrent pregnancy loss across both age groups (≤ 35 years and > 35 years) and reduced early and late miscarriage rates in those ≤ 35 years. However, its benefits diminished in patients undergoing fresh embryo transfers, suggesting that the embryo transfer strategy may influence its effectiveness.

## 1. Background

Pregnancy loss is a distressing complication that affects 15%–25% of clinically recognized pregnancies, with nearly 80% of losses occurring in the first trimester [1]. A more severe manifestation, recurrent pregnancy loss (RPL), impacts 1%–5% of women and is defined as the loss of two or more consecutive or nonconsecutive pregnancies. It includes biochemical pregnancies and pregnancies of unknown location [1, 2]. RPL carries significant physical and psychological consequences. Beyond clinical risks such as infection and hemorrhage, affected individuals often experience profound psychological distress, including anxiety, depression, and so on [3].

The etiologies of RPL are diverse and multifactorial. Well‐established causes include parental chromosomal rearrangements (e.g., balanced translocations) [4], uterine structural anomalies [5], endocrine dysfunctions [6], and autoimmune conditions like antiphospholipid syndrome [7]. Despite advances in diagnostic protocols, over 50% of RPL cases remain unexplained, particularly when products of conception (POC) are not subjected to cytogenetic analysis [1, 8].

When POC analysis is performed, de novo aneuploidy is identified as a primary etiology in 40%–50% of early pregnancy losses [8, 9]. In this context, preimplantation genetic testing for aneuploidy (PGT‐A) has emerged as a potential strategy to enhance reproductive outcomes in RPL by enabling the selection of euploid embryos, thereby reducing miscarriage risks [8].

Recent advances in molecular diagnostics, including array comparative genomic hybridization (aCGH), digital polymerase chain reaction (dPCR), single‐nucleotide polymorphism (SNP) arrays, real‐time quantitative PCR (qPCR), and next‐generation sequencing (NGS), have significantly improved the accuracy of PGT‐A [10]. Trophectoderm (TE) biopsy at the blastocyst stage, combined with comprehensive chromosome screening (CCS), provides a superior alternative to cleavage‐stage biopsy by overcoming limitations such as incomplete chromosomal analysis and reduced live birth rates [11]. Although PGT‐A has shown promise in reducing miscarriage rates, especially in women of advanced maternal age (AMA), its efficacy in RPL remains controversial. Several studies report improved outcomes following PGT‐A in RPL populations [12, 13], particularly among those with three or more losses. In contrast, other investigations have found no significant benefit. For instance, a meta‐analysis by Mastenbroek et al. [14] revealed that PGT‐A might adversely affect live birth rates in AMA. Further, randomized controlled trials (RCTs) demonstrated no improvement in ongoing pregnancy or live birth rates in women under 35 years of age [15, 16] Importantly, many of these studies evaluated only the first embryo transfer (ET) as the primary outcome, which may not fully capture the clinical utility of PGT‐A. Current expert consensus emphasizes the cumulative live birth rate (CLBR) per oocyte retrieval cycle as a more comprehensive and patient‐centered outcome measure in IVF [17].

Given the above discordant findings, the study is aimed at evaluating reproductive outcomes in women with RPL undergoing IVF with or without PGT‐A. The primary outcome is the CLBR per oocyte retrieval cycle. Secondary outcomes include live birth rate per transfer, clinical pregnancy rate, early miscarriage rate, and late miscarriage rate. These findings will provide critical insights for clinicians managing RPL and guide evidence‐based patient counseling [18].

## 2. Methods

### 2.1. Study Design

**Figure 1:**
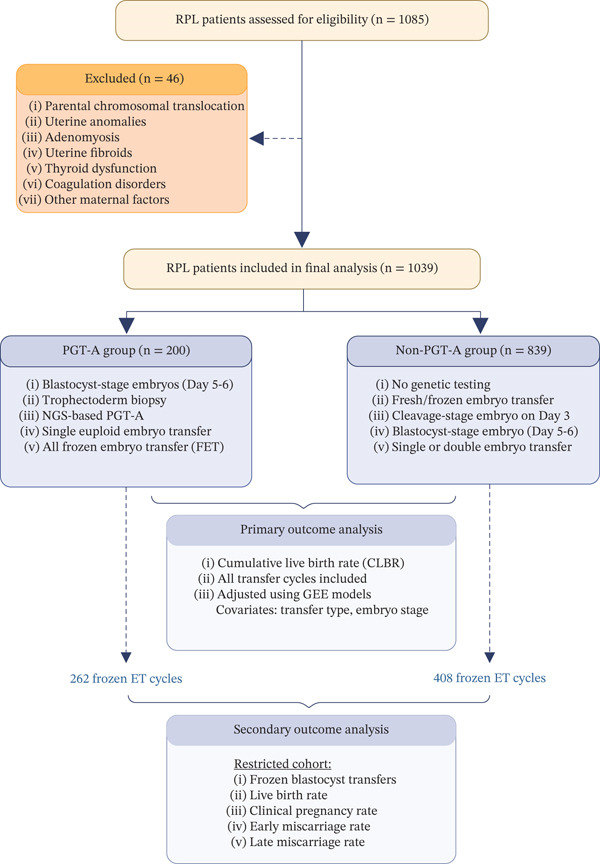
RPL: recurrent pregnancy loss; PGT‐A: preimplantation genetic testing for aneuploidy; FET: frozen embryo transfer; CLBR: cumulative live birth rate; GEE: generalized estimating equation models.

We conducted a retrospective cohort study by searching the medical record database of the Reproductive Medicine Center, Nanjing Drum Tower Hospital to identify infertile women with a medical history of RPL who underwent IVF between January 2013 and December 2023. The process of patient screening, exclusion, group allocation and outcome assessment is summarized in Figure 1. The study included couples with two or more failed clinical pregnancies between 6 and 24 weeks of gestation, primarily between 6 and 12 weeks, excluding ectopic, molar, or biochemical pregnancies (according to the ASRM guidelines). Exclusion criteria included chromosomal abnormalities in either partner, anatomical abnormalities (e.g., uterine malformations such as unicornuate uterus and duplex uterus, untreated septate uterus, adenomyoma, submucosal uterine fibroids, or endometrial polyps), hypothyroidism, thrombophilia, antiphospholipid antibody syndrome (APS), and other severe comorbidities to ensure that the study focused on RPL cases without confounding factors. A total of 1039 couples with RPL (200 who underwent PGT‐A and 839 who did not) met the inclusion criteria and were included in the study; a total of 670 frozen embryo transfer (FET) cycles with a single embryo were included. Outcome data were available for all participants. Patient cycles were identified using consistent identification codes and organized by the date of oocyte retrieval and ET. This study was approved by the Ethics Committee (Approval No. 2021‐384‐01). All patient information was fully anonymized prior to analysis, and data were handled in accordance with institutional data‐protection policies. The workflow of the study is shown in Figure 1.

### 2.2. Ovarian Stimulation, Oocyte Retrieval, Embryo Culture, and PGT‐A

Patients underwent controlled ovarian stimulation (COS), oocyte retrieval, and ET following standard protocols, selected based on physician discretion and individualized according to patient characteristics, including age and ovarian reserve, as estimated by serum anti‐Müllerian hormone (AMH) and/or basal follicle‐stimulating hormone (FSH) levels. Ovarian stimulation was conducted using either long or short protocols with gonadotropin‐releasing hormone (GnRH) agonists, GnRH antagonists, or clomiphene citrate (CC). Oocyte maturation was triggered with 5000 IU of human chorionic gonadotropin (hCG) when the leading follicle exceeded 20 mm in diameter, as measured by transvaginal ultrasonography. Oocyte retrieval was performed 36 hours post‐hCG administration under ultrasound guidance.

In the PGT‐A group, intracytoplasmic sperm injection (ICSI) was used for fertilization. Normal fertilization was confirmed by the presence of two polar bodies (2 PB) and two pronuclei (2 PN) at 16–18‐h postinsemination. Fertilized embryos were cultured under controlled conditions at 37°C in an atmosphere of 5% O_2_ and 6% CO_2_ using a sequential culture medium (G1/G2; Vitrolife, Sweden). Embryos that arrested in development or exhibited > 50% fragmentation by Day 3 were discarded, whereas the remaining embryos were cultured to the blastocyst stage. Blastocysts were evaluated according to the Gardner criteria; Two senior embryologists independently evaluated morphology, with discrepancies resolved by consensus [19]. TE biopsies were performed on Days 5 or 6 for blastocysts with a morphological score of 4 BC or higher using laser technology. Biopsied TE cells underwent whole‐genome amplification using the PicoPLEX WGA kit (Takara, Japan), followed by chromosomal ploidy analysis via NGS on the Illumina platform at approximately 0.05× coverage. This approach enables reliable detection of aneuploidies and CNVs ≥ 4 Mb. After biopsy, blastocysts were vitrified using the Kitazato Vitrification Kit (Kitazato, Tokyo, Japan). In the non‐PGT‐A group, either conventional in vitro fertilization (IVF) or ICSI was performed, depending on the presence or absence of male factor infertility. Embryos were either transferred fresh (1–2 embryos) on Day 3 or Day 5/6, or underwent FET in subsequent cycles.

### 2.3. Clinical Outcome and Statistical Analysis

The primary outcome was the CLBR, which included all COS cycles and up to three ETs. Secondary outcomes were assessed only in single‐embryo FET cycles (*n* = 670) and included the clinical pregnancy rate, live birth rate, early miscarriage rate, and late miscarriage rate per ET. Biochemical pregnancy was defined as a positive serum hCG (> 100 mIU/mL) on Day 14 post‐ET without ultrasound evidence of a gestational sac. Clinical pregnancy was confirmed by the detection of a gestational sac and fetal heartbeat via transvaginal ultrasonography at 4 and 6 weeks after transfer (approximately 7 and 9 weeks of gestation). Early miscarriage was defined as the loss of a previously detected fetal heartbeat before 12 weeks of gestation, whereas late miscarriage was defined as the loss of an intrauterine clinical pregnancy between 12 and 28 weeks of gestation.

Continuous variables were presented as mean ± standard deviation (SD) or median (range) depending on data distribution, whereas categorical variables were expressed as counts and percentages. The Student′s *t*‐test or the Mann–Whitney *U*‐test was used for parametric and nonparametric continuous variables, respectively. The chi‐square test or Fisher′s exact test was applied to categorical variables, as appropriate. To account for potential clustering of multiple treatment cycles within the same couple, generalized estimating equation (GEE) models were applied where applicable. Multivariable regression analyses were used to estimate adjusted relative risks (aRRs) and 95% confidence intervals (CIs) for reproductive outcomes. Covariates considered in adjusted models included maternal age, body mass index (BMI), basal FSH level, antral follicle count (AFC), number of previous pregnancy losses, duration of infertility, and use of PGT‐A. Variables significantly associated with outcomes in univariable analyses (*p* < 0.05) were retained in the final models. Given the significant effect of maternal age on embryo euploidy (Cimadomo et al. 2021), patients were stratified into two groups based on age: ≤ 35 years (younger patients) and > 35 years (AMA), and analyzed separately for PGT‐A and non‐PGT‐A groups. All statistical analyses were performed using SPSS software (Version 21; IBM Corporation, New York, United States), with a two‐sided *p* value < 0.05 considered statistically significant.

## 3. Results

### 3.1. Baseline Characteristics of RPL Patients

A total of 1039 couples met the inclusion criteria described in the materials and methods, including 200 women with 230 COS cycles in the PGT‐A group and 839 women with 1192 COS cycles in the non‐PGT‐A group. Data for these cycles were retrospectively analyzed for this study (Table 1). No significant differences were observed between the PGT‐A and non‐PGT‐A groups in terms of maternal age, paternal age, maternal BMI, number of pregnancy losses, basal reproductive hormone levels (including FSH, luteinizing hormone [LH], prolactin [PRL], estradiol [E_2_], testosterone [T]), and AFC. However, the duration of infertility was significantly longer in the non‐PGT‐A group.

**Table 1:** Baseline characteristics of PGT‐A and non‐PGT‐A groups.

	PGT‐A group	Non‐PGT‐A group	*p*
Number of infertile couples (*n*)	200	839	
Maternal age (year, $\bar{x}\pm \text{SD}$)	33.73 ± 4.56	34.05 ± 5.17	0.409
Paternal age (year, $\bar{x}\pm \text{SD}$)	34.69 ± 5.05	35.50 ± 6.04	0.055
Maternal BMI (kg/m^2^, $\bar{x}\pm \text{SD}$)	22.62 ± 2.73	23.35 ± 7.13	0.086
Duration of infertility years (year, median ± IQR)	1.00 [1.00, 2.00]	2.00 [1.00, 3.00]	< 0.001
Pregnancy loss (*n*, median ± IQR)	2.00 [2.00, 3.00]	2.00 [2.00, 3.00]	0.172
Female reproductive endocrine hormones^a^			
FSH (mIU/mL, $\bar{x}\pm \text{SD}$)	7.50 ± 3.40	7.84 ± 3.16	0.165
LH (mIU/mL, $\bar{x}\pm \text{SD}$)	5.19 ± 2.65	5.27 ± 3.43	0.689
PRL (ng/mL, $\bar{x}\pm \text{SD}$)	25.59 ± 42.64	29.62 ± 68.91	0.307
E_2_ (pg/mL, $\bar{x}\pm \text{SD}$)	49.00 ± 71.74	50.68 ± 88.65	0.781
T (ng/dL, $\bar{x}\pm \text{SD}$)	0.68 ± 3.20	1.02 ± 6.10	0.282
AFC (*n*, $\bar{x}\pm \text{SD}$)	15.78 ± 9.62	14.59 ± 8.05	0.068

Abbreviations: AFC, antral follicle count; BMI, body mass index; E_2_, estradiol; FSH, follicle‐stimulating hormone; IQR, interquartile range; LH, luteinizing hormone; PGT‐A, preimplantation genetic testing for aneuploidy; PRL, prolactin;T, testosterone; SD, standard deviation.

^a^Menstrual day 3–5 basal endocrine hormones.

### 3.2. Characteristics of the Two Groups in Ovarian Stimulation and Embryo Development

The PGT‐A group underwent a total of 230 COS cycles. Among them, 177 (88.50%) patients underwent COS once, 17 (8.50%) twice, and five (2.50%) and one (0.50%) undergoing COS three and four times, respectively. In comparison, the non‐PGT‐A group underwent 1192 COS cycles, with 623 (74.26%) undergoing COS once, 141 (16.81%) twice, and 42 (5.01%), 16 (1.91%), and 17 (2.03%) undergoing COS three, four, and five or more times, respectively (Table 2). The duration of gonadotropin administration and total gonadotropin dosage were comparable between the two groups (Table 2). The PGT‐A group had significantly higher numbers of retrieved oocytes, MII oocytes, fertilized oocytes, and maximum estrogen levels on the day of hCG administration compared with the non‐PGT‐A group (Table 2). However, the number of euploid embryos available for transfer after PGT‐A testing was significantly lower in the PGT‐A group (3.09 ± 1.68 vs. 5.44 ± 3.02, *p* < 0.001). Endometrial thickness was comparable between the groups. (Table 2).

**Table 2:** COS, oocyte retrieval, and embryo development of PGT‐A and non‐PGT‐A groups.

	PGT‐A group	Non‐PGT‐A group	*p*
Number of COS cycles (*n*)	230	1192	
Number of infertile couples (*n*)	200	839	
Number of COS cycles for patients, *n* (%)			
1	177 (88.50)	623 (74.26)	< 0.001
2	17 (8.50)	141 (16.81)	< 0.001
3	5 (2.50)	42 (5.01)	< 0.001
4	1 (0.50)	16 (1.91)	< 0.001
≥ 5	0	17 (2.03)	< 0.001
Gonadotropin days ($\bar{x}\pm \text{SD}$)	9.66 ± 3.07	10.03 ± 3.66	0.069
Gonadotropin dosage (IU, $\bar{x}\pm \text{SD}$)	1994.28 ± 840.86	1923.11 ± 942.43	0.192
Maximum estrogen level on day of hCG (pg/mL, $\bar{x}\pm \text{SD}$)	3,594.35 ± 2556.38	2,737.88 ± 2233.20	<0.001
Retrieval oocytes (*n*, $\bar{x}\pm \text{SD}$)	13.85 ± 6.67	9.60 ± 6.15	< 0.001
MII oocytes (*n*, $\bar{x}\pm \text{SD}$)	8.57 ± 6.66	7.34 ± 5.70	0.003
Normal fertilized oocytes (*n*, $\bar{x}\pm \text{SD}$)	7.34 ± 5.92	6.14 ± 4.97	0.001
Embryos available for transfer (*n*, $\bar{x}\pm \text{SD}$)	3.09 ± 1.68	5.44 ± 3.02	< 0.001
Endometrial thickness (mm, $\bar{x}\pm \text{SD}$)	8.73 ± 1.62	9.00 ± 2.91	0.582

Abbreviations: COS, controlled ovarian stimulation, aneuploidy; hCG, human chorionic gonadotrophin; MII, Metaphase II.

### 3.3. CLBR Was Higher in the PGT‐A Group

Given the significant impact of maternal age on embryo euploidy and the need to adjust for covariates, patients were stratified into two age groups: ≤ 35years and > 35 years. Among women diagnosed with RPL, the application of PGT‐A was significantly associated with an increased CLBR, at 58% versus 38% (a*RR*: 1.52, 95% CI [1.19–1.93], *p* = 0.001), regardless of age (Table 3). Stratified by age, CLBR was 70% versus 51% (a*RR*: 1.38, 95% CI [1.05–1.82], *p* = 0.025) for women ≤ 35 years and 35% vs. 21% (a*RR*: 1.69, 95% CI [1.03–2.77], *p* = 0.037) for those > 35 years. (Table 3).

**Table 3:** Comparison of cumulative live birth rate between PGT‐A and non‐PGT‐A groups.

	PGT‐A group	Non‐PGT‐A group	*p*	a*RR* (95% CI)
Cumulative live birth rate (%)				
Total	134/230 (58.26%)	458/1192 (38.42%)	0.001	1.52 (1.19, 1.93)
≤ 35	108/155 (69.68%)	359/710 (50.56%)	0.025	1.38 (1.05, 1.82)
> 35	26/75 (34.67%)	99/482 (20.54%)	0.037	1.69 (1.03, 2.77)

Abbreviations: a*RR*, adjusted risk ratio; COS, controlled ovarian stimulation.

Secondary outcomes, including the live birth rate (LBR), clinical pregnancy rate, early miscarriage rate, and late miscarriage rate per ET, were evaluated only in single‐embryo FET cycles (*n* = 670) and are presented for the PGT‐A and non‐PGT‐A groups in Table 4. Notably, the results indicated that the application of PGT‐A significantly increased the LBR, from 31% to 51% (*p* < 0.001), regardless of age. When stratified by age, LBR was 54% versus 41% (*p* = 0.007) for women ≤ 35 years and 41% versus 18% (*p* < 0.001) for women > 35 years. In addition, PGT‐A also significantly increased the clinical pregnancy rate from 48% to 59% (*p* = 0.006), regardless of age. Furthermore, in patients ≤ 35 years, PGT‐A reduced the miscarriage rate. The early miscarriage rate decreased from 14% to 7% (*p* = 0.028), and the late miscarriage rate decreased from 3% to 0% (*p* = 0.032).

**Table 4:** Comparison of clinical outcomes between PGT‐A and non‐PGT‐A groups in single‐embryo FET cycles.

	PGT‐A group	Non‐PGT‐A group	*p*
Number of ET cycles (*n*)	262	408	
Clinical pregnancy rate (%)			
Total	155/262 (59.16%)	196/408(48.04%)	0.006
≤ 35	124/199 (62.31%)	135/229(58.95%)	0.489
> 35	31/63 (49.21%)	61/179(34.08%)	0.036
Live birth rate (%)			
Total	134/262 (51.15%)	126/408(30.88%)	< 0.001
≤ 35	108/199 (54.27%)	94/229(41.05%)	0.007
> 35	26/63 (41.27%)	32/179(17.88%)	< 0.001
Early miscarriage rate (≤ 12w) (%)			
Total	17/262 (6.49%)	57/408(13.97%)	0.002
≤ 35	14/199 (7.04%)	32/229(13.97%)	0.028
> 35	3/63 (4.76%)	25/179(13.97%)	0.065
Late miscarriage rate (≤ 12w) (%)			
Total	0/262 (0.00%)	8/408(1.96%)	0.026
≤ 35	0/199 (0.00%)	6/229(2.62%)	0.032
> 35	0/63 (0.00%)	2/179(1.12%)	1

Abbreviation: ET, embryo transfer.

## 4. Discussion

Our study demonstrates that the application of preimplantation genetic testing for aneuploidy (PGT‐A) in women with recurrent pregnancy loss (RPL) significantly improves CLBR while concurrently reducing both early and late miscarriage rates. These findings align with previous studies that have highlighted the benefits of PGT‐A in this specific patient population [12, 13]. Importantly, women who underwent FET following PGT‐A exhibited higher live birth and clinical pregnancy rates, further reinforcing the value of incorporating genetic screening into assisted reproductive strategies for RPL.

Fresh ET is often offered to patients with favorable prognoses—those who respond well to ovarian stimulation and have multiple high‐quality embryos available. This makes CLBR, which reflects all ETs from a single ovarian stimulation cycle, a more reliable and patient‐centered primary outcome in evaluating IVF success [17]. Our findings reinforce the value of the CLBR rate as the primary endpoint in clinical trials, whereas current ASRM guidelines do not recommend routine PGT‐A for all IVF cycles due to insufficient evidence of benefit across all patient groups [1]. Indeed, prior studies have shown that in women with favorable prognoses, including younger women and those with good‐quality embryos, conventional IVF can achieve CLBR comparable to those with PGT‐A, irrespective of maternal age [20]. However, our results suggest that the benefits of PGT‐A become more pronounced in women with RPL, a population often characterized by lower implantation potential and higher miscarriage risk. In both younger (≤ 35 years) and older (> 35 years) subgroups, PGT‐A was associated with a significant increase in CLBR. Notably, this improvement was achieved despite fewer embryos being transferred per cycle in the PGT‐A group, which contributed to a lower incidence of multiple pregnancies. These outcomes underscore the dual advantage of PGT‐A in enhancing singleton pregnancy outcomes while minimizing risks associated with multiple gestations.

The core rationale for PGT‐A lies in its capacity to screen embryos for aneuploidy prior to implantation, thus improving embryo selection and reducing early pregnancy loss. Although several variables have been evaluated for their association with embryo euploidy rates—including prior reproductive history, IVF outcomes, and implantation failures—maternal age at oocyte retrieval remains the most consistent predictor [21]. Interestingly, a recent study reported that younger women (≤ 35 years) with idiopathic RPL exhibit a disproportionately high incidence of chromosomal abnormalities in blastocysts—48.9%, compared with 36.9% in women with sporadic pregnancy loss [22]. Similarly, Chen et al. [20] reported that among women aged 20–37 years, the euploidy rate was approximately 69.8%, whereas in our cohort, women under 35 with RPL had a lower euploidy rate of 58.4%. These findings point to a distinct biological profile in younger RPL patients, suggesting that the prevalence of chromosomal abnormalities may play a more prominent role in recurrent loss than previously recognized. Our results also lend support to the implantation checkpoint hypothesis [23], which posits that successful implantation requires not only a competent embryo but also a receptive and selectively discriminating endometrium. An overly permissive endometrium may allow the implantation of aneuploid embryos, subsequently resulting in early pregnancy loss. PGT‐A, by promoting euploid embryo selection, may optimize this dynamic and improve reproductive efficiency. Consistent with prior studies [20, 24, 25], our findings demonstrate a markedly lower early miscarriage rate in the PGT‐A group—6% overall, with rates of 7% in patients ≤ 35 years and 4% in those > 35 years—well below the expected baseline rate of 15%–25%. These data reinforce the clinical utility of PGT‐A in reducing early pregnancy loss, regardless of maternal age, and enhance the likelihood of progression to viable pregnancy.

Our findings highlight the potential value of tailoring ET strategies to individual patient profiles. In particular, the clinical benefit of PGT‐A appears most evident in FET cycles, suggesting that integrating embryo ploidy assessment with a freeze‐all strategy may optimize outcomes for selected patients. These observations support a more individualized, strategy‐driven approach in ART practice, rather than a uniform application of PGT‐A across all patients and transfer types.

Despite the strengths of this study, several limitations must be acknowledged. First, the retrospective design introduces inherent bias and limits causal inference. Second, the study cohort consists of women with RPL undergoing IVF, which may include indications beyond RPL itself and therefore may not be generalizable to all RPL patients, particularly those not requiring IVF. Furthermore, the PGT‐A group exclusively utilized the FET strategy, whereas the control group included both fresh and frozen transfers, as well as cleavage‐ and blastocyst‐stage transfers. To reduce heterogeneity and potential confounding in the analysis of secondary outcomes, comparisons were restricted to single‐embryo FET cycles, including live birth rate, clinical pregnancy rate, early miscarriage rate, and late miscarriage rate per ET. Nevertheless, some residual confounding may remain, as blastocyst‐stage transfers are independently associated with improved implantation and live birth rates [26].

Importantly, these findings suggest that the potential clinical benefit of PGT‐A may be context‐dependent and most pronounced within an FET‐based treatment framework, rather than uniformly applicable across all ET strategies. This highlights the importance of individualized clinical decision‐making when integrating PGT‐A into ART practice. At the same time, potential safety concerns associated with FET—including hypertensive disorders of pregnancy, higher birth weights, and an increased risk of large‐for‐gestational‐age infants—should be carefully considered when counseling patients [27].

Taken together, our findings contribute important evidence to the ongoing debate regarding the clinical utility of PGT‐A in women with recurrent pregnancy loss. When implemented as part of an IVF strategy involving frozen‐thawed blastocyst transfer, PGT‐A was associated with significantly higher CLBR and lower miscarriage rates, regardless of maternal age. The benefit appears to be particularly relevant in RPL populations, where chromosomal abnormalities are a predominant cause of early pregnancy loss. By facilitating the selection of euploid embryos, PGT‐A may enhance reproductive efficiency and alleviate both the emotional and physical burden of repeated pregnancy loss. However, the lack of observed benefit in fresh transfer cycles and in patients with favorable prognoses emphasizes the need for individualized clinical decision‐making. These findings reinforce the need for personalized treatment strategies based on reproductive history, prognosis, and overall clinical context, and support the selective use of PGT‐A in women with RPL undergoing IVF.

## 5. Conclusions

In conclusion, this study provides evidence that PGT‐A, when applied in the context of IVF for women with recurrent pregnancy loss, can significantly enhance cumulative live birth rate and reduce miscarriage rate. These benefits were observed regardless of maternal age. Nonetheless, the utility of PGT‐A in RPL patients without concomitant infertility remains uncertain. Therefore, although our results support the potential integration of PGT‐A into clinical practice for selected RPL cases, larger, well‐designed randomized controlled trials, including prospective multicenter validation studies, are needed to confirm its efficacy and to inform individualized treatment strategies.

## Author Contributions

L.Y. and Y.T. participated in study design, analysis, manuscript drafting, and critical discussion. N.K., J.L., F.H., and N.Z. participated in study analysis, execution, statistical analysis, and critical discussion. J.M. participated in study design, execution, statistical analysis, manuscript drafting, and critical discussion. L.Y. and Y.T. are co‐first authors.

## Funding

This study was supported by the National Natural Science Foundation of China (10.13039/501100001809) (82301891, 82371678).

## Disclosure

All authors read and approved the final manuscript. A preprint of this article has previously been published [28].

## Ethics Statement

This study was approved by the Ethics Committee of Nanjing Drum Tower Hospital (Approval No. 2021‐384‐01). Written informed consent was obtained from all participants prior to enrollment.

## Consent

The authors have nothing to report.

## Conflicts of Interest

The authors declare no conflicts of interest.

## Data Availability

The datasets used and/or analyzed during the current study are available from the corresponding author on reasonable request.
